# Successful Management of an Acetabular Fracture Associated With Comorbidities in an 80-Year-Old Patient: A Case Report

**DOI:** 10.7759/cureus.67656

**Published:** 2024-08-24

**Authors:** Ghassan A Alnajjar, Ahmad Ezzelden, Mostafa Elgendy, Fahad s Abbas

**Affiliations:** 1 Orthopedic Surgery, Saudi German Hospital, Jeddah, SAU

**Keywords:** pelvic-acetabulum, open reduction internal fixation, geriatric injuries, acetabular fractures, elderly trauma

## Abstract

Pelvic fractures, particularly acetabular fractures, pose major problems for individuals with advanced age due to comorbidities and poor bone quality. Road traffic accidents (RTAs) are a leading cause of high-energy injuries. This case report describes the treatment of an 80-year-old patient with hypertension, pulmonary fibrosis, and morbid obesity who suffered an acetabular fracture after an RTA. An 80-year-old patient was received in the emergency room 10 days after the RTA. X-rays and CT scans indicated an anterior column with a posterior hemi-transverse fracture, and the quadrilateral plate was completely displaced. A CT angiography revealed deep vein thrombosis (DVT) in the lower limb, prompting the start of anticoagulant medication and the insertion of an inferior vena cava (IVC) filter. The modified Stoppa technique was used to definitively fix the acetabular fracture. The corona mortis was found and safeguarded throughout the surgery. Following surgery, the patient avoided weight-bearing activities for one month before beginning non-weight-bearing mobilization and physical therapy. At the first clinic visit following discharge, the patient reported total pain relief and successful mobilization with a wheelchair. The treatment of acetabular fractures in older patients with comorbidities necessitates a specialized, multidisciplinary approach. This case indicates that, despite major hurdles, successful outcomes can be achieved through appropriate surgical and postoperative techniques. Future research should focus on refining these methods to enhance the prognosis in this patient population.

## Introduction

The incidence of pelvic fractures is increasing due to the aging population [1]. The increasing prevalence of osteoporosis has created considerable hurdles in the management of injuries in patients with advanced age [2]. Historically, high-energy pelvic fractures associated with significant trauma predominated; however, the prevalence of low-energy fragility fractures is growing and is expected to rise further in the future [1,2]. A prime cause of such injuries involving lower extremities is road traffic accidents (RTA). Men aged more than 70 are more affected, and the common fractures involve the femoral neck [3]. Acetabular fractures are high-energy injuries often resulting from high-speed car crashes, falls from heights, and extreme sporting events [3,4]. The incidence of these fractures has remained stable at three per 100,000 people per year [3]. Pathophysiology involves the impact of the femoral head on the articular surface, with anterior and posterior fracture patterns depending on the hip's position. Initial assessment involves trauma assessment and resuscitation protocols, with a complete review of the musculoskeletal system, peripheral nerves, and skin [5]. Diagnosis is possible with plain X-rays, but CT scans are often necessary due to multiple organ injuries. Plain films with an AP pelvis and Judet views are often obtained first, with six radiographic landmarks identified on an AP view of the pelvis [3]. Management of acetabular fractures in patients with associated comorbidities is particularly challenging. Late hospital presentation may also determine management decisions [3-5]. The main purpose of treatment is to preserve the hip joint anatomy and blood supply to the femoral head while quickly and completely allowing the patient to start partial weight bearing or range of motion. Hence, a satisfactory long-term outcome, especially in severe associated comorbidities, could be expected if an accurate diagnosis with timely, proper, and sequential management is made.

This case report details the management of an 80-year-old male with a history of hypertension, lung fibrosis, and morbid obesity who sustained a severe acetabular fracture following an RTA.

## Case presentation

**Figure 1:**
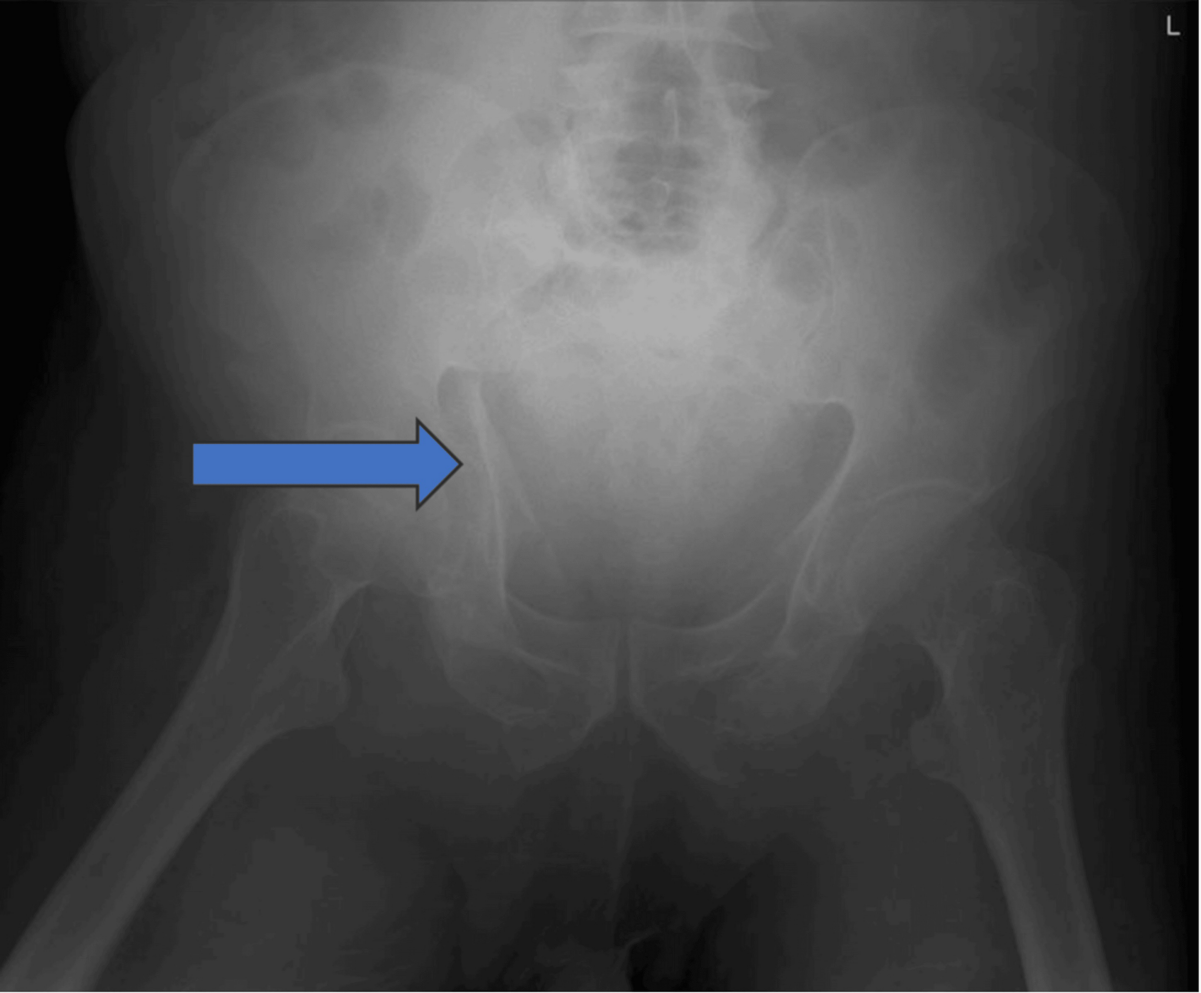
Preoperative X-ray showing disruption of both iliopectineal and ilioischial lines and a disrupted acetabular roof

**Figure 2:**
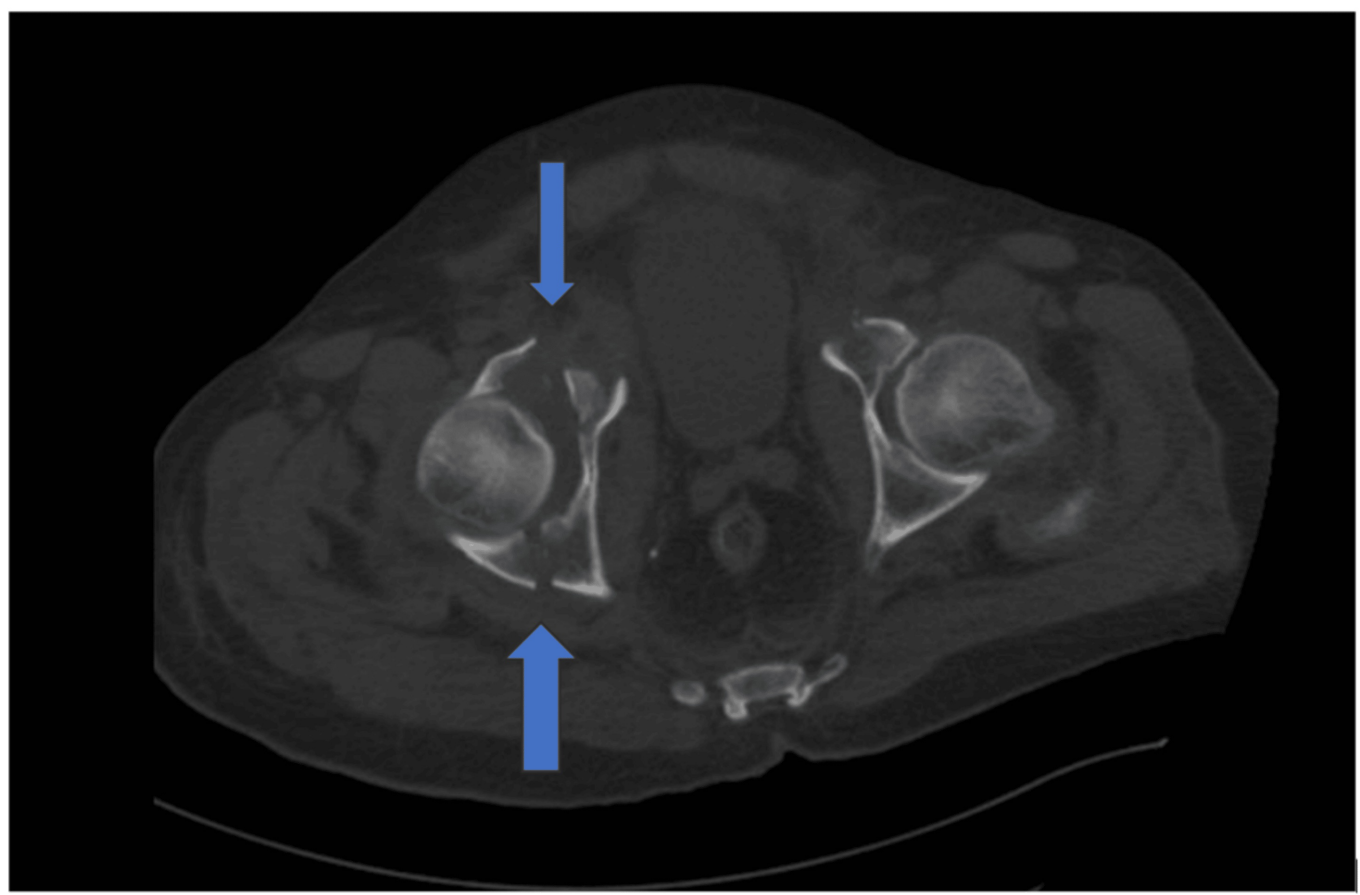
CT scan cut (axial) showing the anterior and posterior wall fractures of the right acetabulum

We present the case of an 80-year-old male with a medical history of hypertension, lung fibrosis, and morbid obesity. The patient presented to the emergency room (ER) 10 days post-injury following an RTA. Upon arrival, X-rays and a trauma CT scan revealed an anterior column with a posterior hemi-transverse fracture and complete displacement of the quadrilateral plate, resulting in the femoral head being completely uncovered (Figures 1-2).

A CT angiogram was subsequently performed, which indicated the presence of deep vein thrombosis (DVT) in the patient's lower limb. Consequently, anticoagulation therapy was initiated. The patient was then taken to the operating room (OR) for the insertion of an inferior vena cava (IVC) filter by the vascular surgery team.

**Figure 3:**
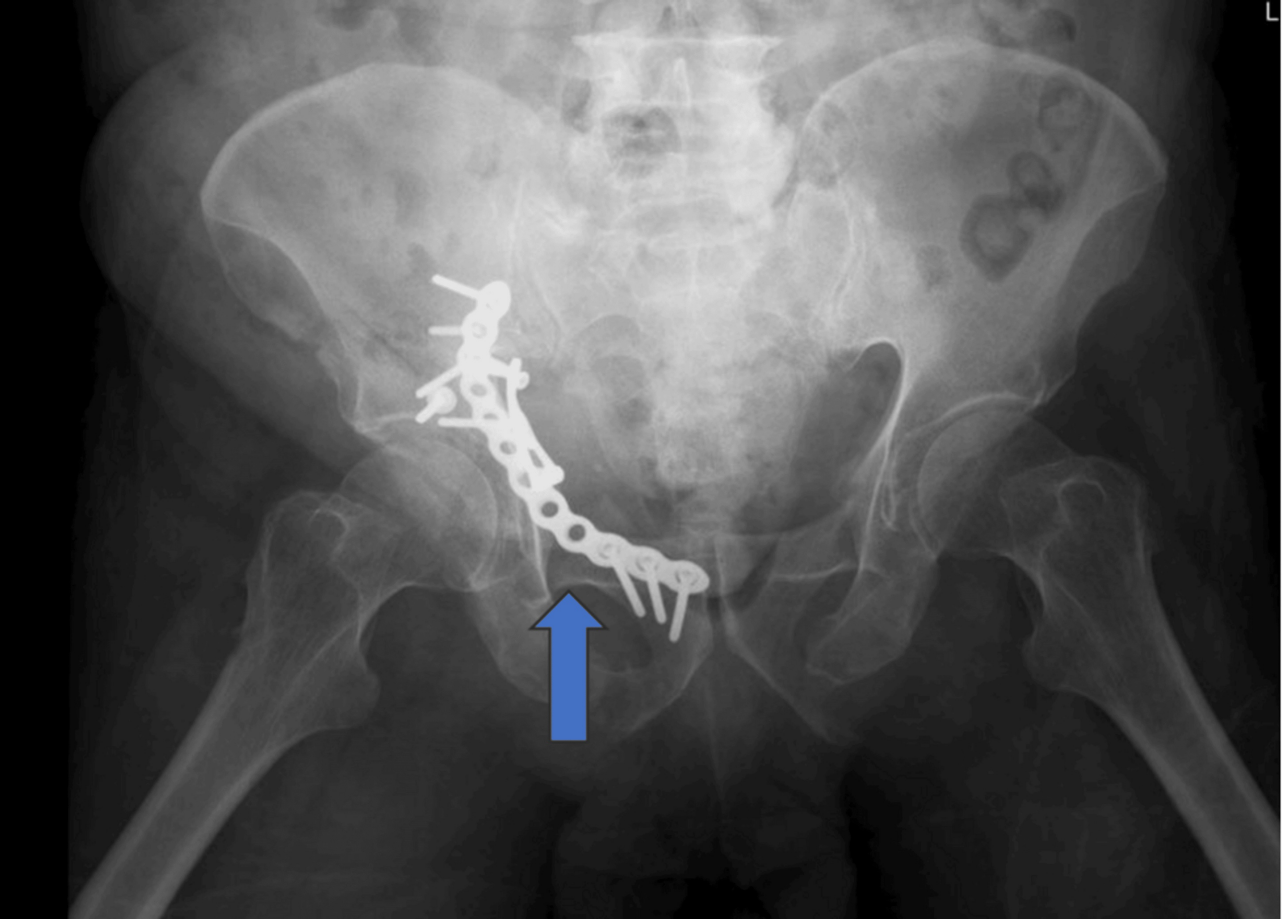
Immediate postoperative X-ray showing that the acetabulum is reduced with an anatomical plate

**Figure 4:**
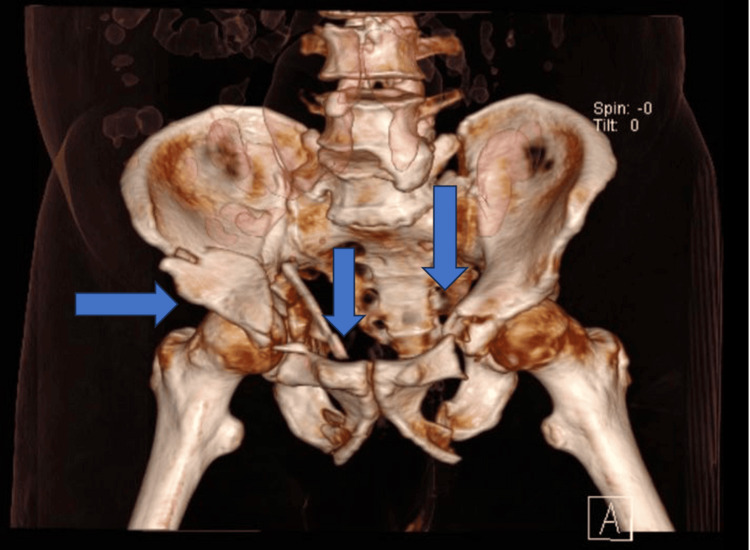
Another cut from a 3D CT scan showing the acetabular fracture along with bilateral superior and inferior pubic rami fractures

One week later, the patient underwent a definitive fixation of the acetabular fracture. The anterior intra-pelvic approach (modified Stoppa approach) was utilized to access the fracture site. During the procedure, the corona mortis was identified and protected. Despite the presence of significant adhesions, they were successfully released without complications. Fracture reduction was achieved under X-ray imaging, and fixation was performed using an anatomical plate (Figure 3). Figure 4 presents another cut from a 3D CT scan showing the acetabular fracture along with bilateral superior and inferior pubic rami fractures.

Postoperatively, the patient was advised to avoid weight-bearing for the first month. At the first clinic visit after discharge, the patient reported complete resolution of pain and was mobilizing with the aid of a wheelchair without bearing weight. After one month, the patient began non-weight-bearing mobilization and started physiotherapy.

## Discussion

This case report describes the successful management of an acetabular fracture in an 80-year-old male who had multiple comorbidities, including high blood pressure, lung fibrosis, and morbid obesity following the RTA. This patient had a complex medical history, and the nature of the injury called for multidisciplinary treatment. Acetabular fractures are severe injuries that generally result from high-energy trauma such as RTAs. For elderly people with osteoporosis and other morbidities, these fractures are more challenging due to poor bone quality and a higher risk for complications. Previous studies have shown that non-operative treatment causes poor results, including chronic pain and disability among elderly patients [6,7]. Therefore, operative management is usually preferred in order to restore joint function and improve quality of life. In our case, definitive fixation was chosen over conservative treatment due to the severity of the fracture and the patient's overall health status.

The modified Stoppa approach used in this case has increasingly become popular because it offers excellent visualization of the quadrilateral plate while limiting surgical morbidity [8]. This approach has been found effective in achieving anatomical reduction while minimizing complications compared with traditional procedures such as Kocher-Langenbeck or ilioinguinal methods [9,10]. The modified Stoppa approach is particularly useful in older adults since limiting tissue damage during surgery is very crucial. Additionally, the use of this approach in our case is consistent with findings from previous studies, which support its use to treat complex acetabular fractures due to its higher advantageous exposure and reducing capacity [8,10,11].

During surgery, it is important to identify corona mortis vessels, as we did, since failing to do so leads to significant bleeding, a common complication noted in the literature [12,13]. The need to appreciate this vascular structure to prevent major intra-operative complications may have resulted in a successful operation.

Following surgery, all patients require close attention, primarily the elderly with associated comorbidities. The use of the IVC filter in our patient was due to DVT, implying that we should not overlook thromboembolic prophylaxis. The addition of an IVC filter is indicated in patients who cannot be anticoagulated with venous thromboembolism or have a high risk of pulmonary embolism, even though most can be managed on anticoagulation alone [14]. Our pre-emptive approach could have helped us avoid more life-threatening thromboembolic events and enabled the patient to recover well.

Early mobilization helps reduce complications such as pneumonia, pressure sores, and venous thromboembolism, which are common among elderly people [15,16]. Initially, our patient was allowed to move without weight bearing, followed by non-weight-bearing physiotherapy after one month, which was consistent with postoperative guidelines. This kind of staged rehabilitation facilitates pain control and the gradual return of mobility.

The positive outcome seen in this case is remarkable, considering that it involved a geriatric patient with multiple comorbidities. Other studies have shown that older adults suffering acetabular fractures tend to do worse compared to younger populations due to factors such as decreased physiological reserve and higher morbidity burden [6,7,17]. Nonetheless, multidisciplinary care involving meticulous preplanning by the surgeon, advanced surgical techniques, and complete postoperative support can help significantly improve outcomes, as evidenced by our case. The results of our case management are in accordance with those of Tannast et al. [18], who found that anatomical reduction and stable fixation were important determinants for good outcomes in acetabular fractures regardless of the patient’s age among 810 patients with acetabular fractures managed surgically within the 26-year period. Our approach to care highlights that we consider a number of factors, such as the overall health status, comorbidities, and specific injury characteristics.

## Conclusions

The management of pelvic fractures in the elderly population remains challenging, and this case report emphasizes an individualized interdisciplinary approach to the management of elderly people with significant comorbidities suffering from complex bone fractures. Our successful utilization of the modified Stoppa technique, along with vigilant thromboembolic prophylaxis, and rehabilitation, further shows that, despite high-risk status, there may be improved outcomes. This highlights that it is crucial to address the patient's various health problems to optimize their health pre-operatively since it raises the patient's probability of survival and improves the outcome of the procedure. Future research should continue exploring better surgical techniques as well as postoperative care protocols aimed at enhancing prognosis in these challenging patient populations.

## References

[1] Wilson DG, Kelly J, Rickman M (2021). Operative management of fragility fractures of the pelvis - a systematic review. BMC Musculoskelet Disord.

[2] Rollmann MF, Herath SC, Kirchhoff F (2017). Pelvic ring fractures in the elderly now and then - a pelvic registry study. Arch Gerontol Geriatr.

[3] Hoge S, Chauvin BJ (2023). Acetabular fractures. StatPearls.

[4] Ferguson TA, Patel R, Bhandari M, Matta JM (2010). Fractures of the acetabulum in patients aged 60 years and older: an epidemiological and radiological study. J Bone Joint Surg Br.

[5] Fischer H, Maleitzke T, Eder C, Ahmad S, Stöckle U, Braun KF (2021). Management of proximal femur fractures in the elderly: current concepts and treatment options. Eur J Med Res.

[6] Carroll EA, Huber FG, Goldman AT, Virkus WW, Pagenkopf E, Lorich DG, Helfet DL (2010). Treatment of acetabular fractures in an older population. J Orthop Trauma.

[7] Shah N, Gill IP, Hosahalli Kempanna VK, Iqbal MR (2020). Management of acetabular fractures in elderly patients. J Clin Orthop Trauma.

[8] Kim HY, Yang DS, Park CK, Choy WS (2015). Modified Stoppa approach for surgical treatment of acetabular fracture. Clin Orthop Surg.

[9] Cole JD, Bolhofner BR (1994). Acetabular fracture fixation via a modified Stoppa limited intrapelvic approach. Description of operative technique and preliminary treatment results. Clin Orthop Relat Res.

[10] Sagi HC, Afsari A, Dziadosz D (2010). The anterior intra-pelvic (modified Rives-Stoppa) approach for fixation of acetabular fractures. J Orthop Trauma.

[11] Ma K, Luan F, Wang X (2013). Randomized, controlled trial of the modified Stoppa versus the ilioinguinal approach for acetabular fractures. Orthopedics.

[12] Noussios G, Galanis N, Chatzis I, Konstantinidis S, Filo E, Karavasilis G, Katsourakis A (2020). The anatomical characteristics of corona mortis: a systematic review of the literature and its clinical importance in hernia repair. J Clin Med Res.

[13] Elhence A, Gupta A (2023). Corona mortise- anatomical variants and implications in pelvic-acetabular surgery: an evidence based review. J Orthop.

[14] DeYoung E, Minocha J (2016). Inferior vena cava filters: guidelines, best practice, and expanding indications. Semin Intervent Radiol.

[15] Ritschel M, Kuske S, Gnass I (2021). Assessment of patient-reported outcomes after polytrauma - instruments and methods: a systematic review. BMJ Open.

[16] Tazreean R, Nelson G, Twomey R (2022). Early mobilization in enhanced recovery after surgery pathways: current evidence and recent advancements. J Comp Eff Res.

[17] Mohan K, Broderick JM, Raza H, O'Daly B, Leonard M (2022). Acetabular fractures in the elderly: modern challenges and the role of conservative management. Ir J Med Sci.

[18] Tannast M, Najibi S, Matta JM (2012). Two to twenty-year survivorship of the hip in 810 patients with operatively treated acetabular fractures. J Bone Joint Surg Am.

